# Valve-in-Valve Transcatheter Aortic Valve Implantation Versus Redo SAVR for Degenerated Biological Prosthesis: A Narrative Review Stating Our Experience

**DOI:** 10.3390/jcm14207158

**Published:** 2025-10-11

**Authors:** Salvatore Torre, Laura Asta, Adriana Sbrigata, Sebastiano Castrovinci, Enrico Amoncelli, Antonio Segreto, Giuseppe Maria Raffa, Gioachino Agostino Giarratana, Vincenzo Argano, Calogera Pisano

**Affiliations:** 1 Cardiac Surgery Unit, Department of Precision Medicine in Medical Surgical and Critical Area (Me.Pre.C.C.), University of Palermo, 90134 Palermo, Italy; salvatore.torre@policlinico.pa.it (S.T.); adriana.sbrigata@gmail.com (A.S.); sebastiano.castrovinci@policlinico.pa.it (S.C.); antonio.segreto@policlinico.pa.it (A.S.); giuseppe.raffa@unipa.it (G.M.R.); 2 Cardiac Surgery Department, Department of Neuroscience, Imaging and Clinical Sciences, University “G.d’Annunzio” Chieti-Pescara, 66100 Chieti, Italy; laura.asta@unich.it; 3 Mediterranean Institute for Transplantation and Specialized Therapies (ISMETT), IRCCS, 90127 Palermo, Italy

**Keywords:** transcatheter aortic valve implantation, redo surgical aortic valve replacement, degenerated biological prosthesis, valve-in-valve procedure

## Abstract

Surgical aortic valve replacement (SAVR) is still the gold-standard treatment for aortic stenosis. However, the increasing use of biological prostheses, even in young patients, makes Valve-in-Valve (ViV) transcatheter aortic valve implantation (TAVI) an attractive option compared to redo SAVR, thanks to its lower invasiveness and sometimes greater safety. However, there are several technical and anatomical aspects to consider. Therefore, the aim of our review is to examine the main mechanisms responsible for the degeneration of biological prostheses and, subsequently, to analyze the hemodynamic (transvalvular gradients, patient–prosthesis mismatch, paravalvular leakage) and technical (risk of coronary obstruction, prosthetic implantation strategy) aspects that most influence the procedure’s success and long-term outcomes. To this end, we present a case we treated in order to enhance our readers’ experience with this procedure. Currently, ViV TAVI is approved for patients at high surgical risk, but it could become a valid option compared to redo SAVR; however, more clinical trials are needed to better analyze the survival differences between these two procedures. Furthermore, it remains a therapeutic strategy reserved for highly specialized centers due to the technical difficulties involved in its execution.

## 1. Introduction

Transcatheter aortic valve implantation (TAVI) has completely changed aortic stenosis treatment, particularly among the elderly [1,2,3]. According to the current European Society of Cardiology (ESC) guidelines for the management of valvular heart disease, TAVI is recommended in older patients (≥75 years) and it is considered a good alternative to surgery in inoperable and high/intermediate-risk patients [1]. Furthermore, the most recent data available in the literature show a significant improvement in the 5-year outcomes of patients undergoing TAVI in terms of valve-related, procedure-related or heart failure-related death, stroke or rehospitalization in low-risk patients [4,5], although, at present, the gold-standard treatment for aortic stenosis remains surgical aortic valve replacement (SAVR). Current guidelines recommend the use of biological prostheses in patients over 60–65 years of age or in cases where lifelong anticoagulant therapy is contraindicated [1]. However, data from the 2024 STS ACSD (Society of Thoracic Surgeons Adult Cardiac Surgery Database) confirm constant growth in the use of biological prostheses compared to mechanical ones (86.6% in 2023, compared to 86.3% in 2022) [4]. This trend can be explained by the increasingly advanced age of patients undergoing SAVR, by the greater durability of biological prostheses [5], and certainly by the advent of and improvements in the Valve-in-Valve (ViV) technique [3]. However, the ViV procedure must be tailored to each patient due to heterogeneity in the biological prostheses used, characteristics of valvular degeneration and individual anatomical characteristics (e.g., height of the coronary ostia) [6]. Therefore, this review aims to analyze the main hemodynamic and technical aspects that need to be evaluated in order to help operators in their decision-making regarding the ViV procedure.

We report the case of a patient who underwent successful ViV TAVI after previous aortic root replacement with a Biointegral conduit.

## 2. Our Experience

In 2019, a 63-year-old man underwent Bentall operation using a 25 mm Biointegral conduit with left ventricle outflow tract (LVOT) reconstruction, mitral valve repair with St Jude Saddle 32 mm annuloplasty ring and aorto-right atrial fistula closure due to complicated native aortic valve infective endocarditis complicated by aortic root and mitroaortic continuity abscess and an aorto-right atrium fistula. The postoperative course was complicated by implantation of a permanent pacemaker for complete heart block.

In November 2023, the patient was referred to our institution for dyspnea (NYHA (New York Heart Association) Class III) and heart failure complicated by pneumonia caused by severe aortic regurgitation. Transthoracic echocardiography (TTE) revealed structural degeneration of the aortic conduit with severe intraprosthetic regurgitation (VC > 6 mm, AR Volume ≥ 60 mL and EROA by PISA ≥ 30 mm^2^). Transesophageal Echocardiography (TEE) and series blood cultures excluded infective endocarditis. Following Heart Team discussion, considering his high surgical risk (EuroSCORE II 12.36%—STS (Society of Thoracic Surgeons) mortality score 4.03%), the patient was admitted and scheduled for ViV TAVI.

**Figure 1:**
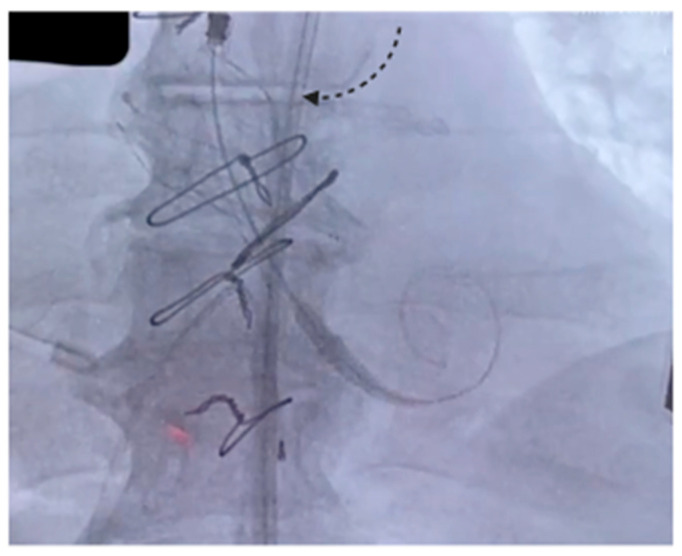
The arrow indicates the expanded prosthesis inside the Biointegral conduit.

The procedure was carried out in a hybrid cardiac surgery theater, under local anesthesia and sedation. A self-expanding Evolut FX Pro 26 mm valve (Medtronic Evolut PRO+, Minneapolis, MN, USA) was implanted through the right common femoral artery. Intraoperative angiography and TEE showed good results and absence of paravalvular leak. The postoperative course was uneventful and TTE showed good results, with a well-functioning prosthesis, a maximum gradient/mean gradient of 18/10 mmHg and no leaks. The patient was discharged on the third postoperative day. Figure 1 shows the self-expanding prosthesis correctly positioned inside the Biointegral conduit.

The complete video of the procedure is available in the Supplementary Materials.

### Bioprosthesis Dysfunction: Main Mechanisms of Degeneration

Bioprosthesis dysfunction represents an important issue considering the increasing rate of bioprosthesis implantation for aortic valve replacement. Bioprosthesis implantation triggers physiopathological processes that lead to its structural deterioration and to specific signs and symptoms based on the valve type, its location and the nature of the complication [7]. The degeneration process could be structural/non-structural and related to thrombosis or endocarditis [8]. Structural valve degeneration is a common, unpreventable and untreatable consequence of bioprosthetic valve implantation. It is often characterized by leaflet calcification and results in stenosis, regurgitation or both [9]. Non-structural bioprosthesis degeneration is due to valve malfunction and is secondary to technical issues occurring during implantation or to a host-mediated response (i.e., pannus formation, suture entrapment, paravalvular leak, valve malpositioning and/or patient–prosthesis mismatch) [9].

Two of the most commonly used materials for biological heart valves (BHVs) are bovine pericardium and native porcine aortic valves. They comprise an extracellular matrix and type I collagen is the predominant protein in both biomaterials [9]. Glutaraldehyde treatment confers structural strength and material durability, stabilizing ultrastructure tissue. Moreover, it reduces, but does not eliminate, bioprosthetic tissue antigenicity. In fact, several studies demonstrate how this treatment causes humoral and cellular immune responses, including infiltration of xenograft by macrophages, T cells and eosinophils [7]. In addition, residual aldehydes have been shown to contribute to calcification of the leaflets [9].

**Figure 2:**
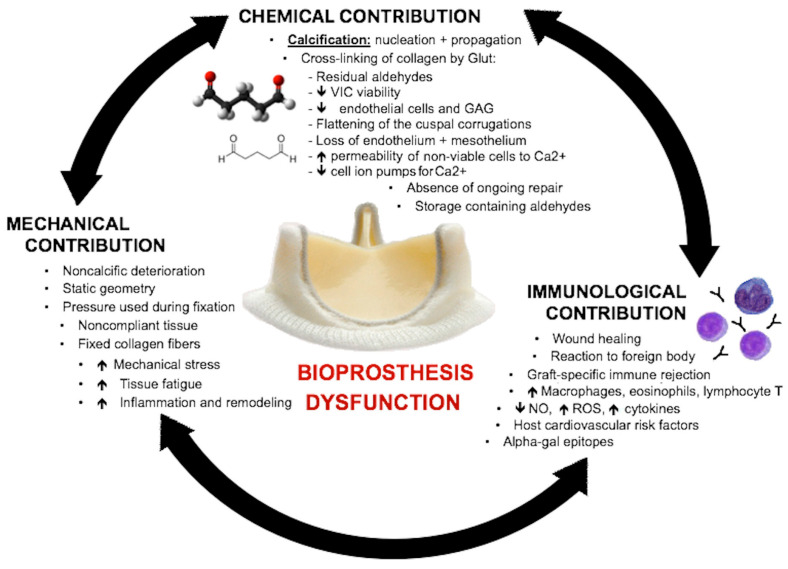
Diagram showing chemical, mechanical and immunological factors that contribute to prosthetic valve dysfunction. GAG = glycosaminoglycans; Glut = glutaraldehyde; NO = nitric oxide; ROS = reactive oxygen species; VIC = valvular interstitial cell [8].

Recent studies have suggested that the deterioration process is multifactorial and mediated by mechanical, chemical and immunological factors (Figure 2). Mechanical and chemical factors act in synergy as pressure–stress and strain–stress, promoting collagen fiber deterioration and, consequently, triggering calcification process. Immunological factors are represented by an innate inflammatory non-specific reaction towards the new foreign body and immune-mediated reaction/inflammation, triggered by exposure of the collagen matrix from which the bioprosthesis is made [9,10]. These reactions decrease nitric oxide (NO) production and increase levels of reactive oxygen species and inflammatory cytokines [8].

Furthermore, an early inflammatory reaction could be triggered by the BioConduit itself, biological fibrin glue and the use of a surgical technique involving Teflon pledget [11].

Galiñanes et al. showed how the No-React BioConduit^®^ graft is seldom associated with foreign-body reactions, with a consequent minor tendency for calcification and deterioration/degeneration. In fact, the Glutaraldehyde treatment in this graft is completed with the use of heparin to lock glutaraldehyde residues, preventing potential immunological reactivity, abolishing its side effects and exclusively keeping its advantages. Moreover, a low infection rate is associated with the No-React BioConduit^®^ graft. Therefore, according to Galiñanes et al., the totally No-React BioConduit^®^ graft may be considered a good alternative to other conduits for aortic root surgery [12].

## 3. ViV-TAVI vs. Redo SAVR: A Literature Review

The advent and constant improvement of TAVI has led to a substantial change in severe aortic stenosis treatment in patients at high and intermediate surgical risk. In parallel, VIV-TAVI was approved by the Food and Drug Administration in March 2015 for the treatment of degenerated bioprostheses [13,14], although it is currently only used in patients at high risk for surgical reintervention.

The STS-ACC TVT Registry (Society of Thoracic Surgeons–American College of Cardiology Transcatheter Valve Therapy Registry) highlights how the number of Viv-TAVI procedures has grown exponentially over the years, from 13,480 procedures in 2013 to 68,511 procedures in 2019 [15]. The most recent reports in the literature, such as the 5-year follow-up from the PARTNER 2 Aortic Valve-in-Valve Registry, continue to stress the positive performance of TAVI in high-risk patients, supported by an improvement in echocardiographic and clinical outcomes [16]. However, the role of the Heart Team remains crucial in evaluating patients and referring them for TAVI or SAVR, as highlighted by American and European guidelines [1,17]. Moreover, we believe that echocardiographic aspects and certain anatomical characteristics deserve further investigation, which is the purpose of our review.

The manuscripts included in our review come from a search for data published in the literature over the last 15 years, performed across the most relevant databases (PubMed, EMBASE, SCOPUS, Web of Science), including only articles written in English based on the inclusion criteria. The literature review was conducted by two different authors (L.A. and C.P.) who independently assessed the inclusion of relevant studies. 

The most relevant articles analysed in this review are summarised in Table 1.

### 3.1. ViV-TAVI vs. Redo SAVR: Hemodynamic Aspects

#### 3.1.1. Transvalvular Gradients

According to the literature, mean transvalvular gradients in ViV-TAVI are higher than in TAVI on native aortic valves: Akodad et al., in their retrospective study on 132 patients (49 undergoing ViV-TAVI and 83 undergoing TAVI on native aortic valves), highlighted a mean gradient > 20 mmHg of 37.5% in the first group vs. 8.4% in the second (*p* value 0.0002) [26]. A mean gradient of 16.9 mmHg emerges at one year of clinical follow-up from the Valve-in-Valve International Data Registry Investigators [27]. These data confirm those documented by the Polish Transcatheter Aortic Valve-in-Valve Implantation (ViV-TAVI) Registry [27], as well as those from the more recent PARTNER 2 Aortic Valve-in-Valve Registry, which show a 5-year mean gradient of 16.8 ± 7.90 mmHg (*p* value < 0.0001) [16].

However, of greatest interest in this review, the same clarity of results is not evident when comparing hemodynamic data between ViV TAVI and redo-SAVR. Nalluri et al., in their systematic review and meta-analysis of six observational studies, which included 594 patients, of whom 255 underwent ViV-TAVI and 339 underwent redo-SAVR, did not highlight statistically significant differences in post-procedural transvalvular gradients between the two groups (OR: 3.05, 95%CI: 0.56–16.54; *p* = 0.20) [18]. On the contrary, Gozdek et al., in their review and meta-analysis study, documented the echocardiographic and hemodynamic superiority of redo-SAVR compared to ViV-TAVI (postoperative aortic valve gradient > 20 mmHg OR 3.66, 95% CI 0.44–30.58) [19]. More recently, Dokollari et al., in an single-center observational cohort study, did not show statistically significant differences in transvalvular gradients (ViV-TAVI 16.8 ± 1 mmHg, redo-SAVR 16.2 ± 7.2 mmHg, *p* = 0.72), although it should be emphasized that the study involved a small population (31 vs. 57 patients in the two respective groups) [20].

The non-univocity of the data present in the literature can be explained by the numerous anatomical and technical aspects that can influence the variability in the hemodynamic function of the implanted prosthesis. The etiology of valvular degeneration, the type and size of the previously implanted prosthesis, the implantation technique and the anatomy of the aortic root are determining factors in influencing the transvalvular gradients [6]. In particular, patients with valvular degeneration due to stenosis have higher mean post-procedural transvalvular gradient values than patients with mixed causes of valvular degeneration or regurgitation, as well as the presence of a small stented surgical aortic valve (<21 mm) (SAV) and the baseline presence of prosthesis–patient mismatch (PPM) immediately after SAVR [3]. To prevent the problem of high gradients after ViV-TAVI, several measures can be adopted: the use of non-compliant balloons to fracture and break the rings of stenotic valves (both as predilation and postdilation with TAVI), or the use of transcatheter valves with supra-annular leaflet attachment. Spaziano et al. have highlighted how the implantation of a prosthesis with supra-annular leaflets, such as the CoreValve, is associated with lower transvalvular gradients compared to prostheses with intra-annular leaflets, such as the Edwards SAPIEN [21].

Post-implant dilatation procedures, and therefore the use of self-expanding and balloon-expandable prostheses, also play a key role. In this regard, a linear regression analysis of the REDUCE registry, which includes patients undergoing ViV-TAVI with the Perimount surgical aortic valve bioprosthesis (Edwards Lifesciences, Irvine, CA, USA), revealed a statistically significant association between the use of self-expanding valves and a reduction in transaortic gradients (−3.25 mmHg, *p* = 0.007) greater than that with balloon-expandable prostheses [28].

#### 3.1.2. Prosthesis–Patient Mismatch

Unlike the results relating to transvalvular gradients, for which there is no univocal interpretation, the frequency of PPM (prosthesis–patient mismatch) is significantly higher in case of ViV-TAVI compared to redo-SAVR. In the Society of Thoracic Surgeons/American College of Cardiology Transcatheter Valve Therapy Registry (TVT Registry), a rate of severe and moderate PPM emerged in 12% and 25% of patients undergoing TAVI, respectively (62,125 patients enrolled between 2014 and 2017). Furthermore, the presence of severe PPM was associated with an increased risk of mortality (OR 1.19, 95% C.I. 1.09–1.31) and hospitalization for heart failure (OR 1.13, 95% C.I. 1.05–1.22) at one year [29].

Raschpichler et al., in a systematic review and meta-analysis of 11 papers published between 2015 and 2021, document the more frequent presence of severe PPM in patients having undergone ViV compared with redo-SAVR (RR, 3.12 [95% CI, 2.35– 4.1], *p* < 0.001) [22]. Similarly, Tam et al. reported the same data emerging from a review and meta-analysis of four unadjusted (n 5298) and two propensity-matched (n 5200) observational studies [23]. In addition to these solid results, it has also been demonstrated that the presence of previous PPM increases the risk of reoperation. In fact, Dauerman et al., in the CoreValve US Expanded Use Study based on 226 patients undergoing ViV-TAVI with very high surgical risk (STS-PROM [Society of Thoracic Surgeons Predicted Risk of Mortality] 9.0 ± 7%), highlighted how the presence of pre-existing PPM was not associated with an increased risk of mortality at 3 years [death rate for no PPM—27.6%, moderate PPM—31.9% and severe PPM—35.2%, in the absence of a statistically significant difference (*p* value = 0.71)]. However, pre-existing PPM was associated with a worsening of quality of life [tested by administration of Kansas City Cardiomyopathy Questionnaire (KCCQ)], as well as a higher rate of reintervention (14.8% vs. 1.6% in moderate PPM vs. 4.4% no PPM, *p* value = 0.02) [30,31].

The motivation behind this scientific evidence is quite clear, and is due to technical differences between the two methods. Surgical valve removal, in addition to the possibility of performing surgical techniques to widen the aortic annulus, significantly reduces the risk of PPM. Conversely, the positioning of another prosthesis in ViV-TAVI further reduces the effective orifice area (EOA), especially if a PPM is already present.

#### 3.1.3. Paravalvular Leak

The presence of paravalvular leaks certainly constitutes the Achilles heel of ViV-TAVI, especially due to the impact that this hemodynamic effect has on medium- and long-term survival [32]. Indeed, it has been widely demonstrated that a greater severity of paravalvular regurgitation is associated with unfavorable medium- and long-term outcomes. In this regard, Sa MP et al., in their meta-analysis of over 25,000 patients, demonstrated how the presence of post-TAVI paravalvular leak negatively impacts the risk of overall mortality (HR, 1.52; 95% CI, 1.43–1.61; *p* < 0.001), rehospitalization (HR, 1.81; 95% CI, 1.54–2.12; *p* < 0.001) and cardiovascular mortality (HR, 1.52; 95% CI, 1.33–1.75; *p* < 0.001) [33].

Since the first TAVI implants, the presence of paravalvular leaks has been one of the least satisfactory hemodynamic aspects. However, technique refinements and prosthesis enhancements have allowed for an improvement in the degree of regurgitation. In particular, the introduction of the Sapien 3 valve (S3: Edwards Lifesciences Inc., Irvine, CA, USA) has improved hemodynamic outcomes, with a reduction in the degree of paravalvular leak compared to the previous Sapien XT (SXT: Edwards Lifesciences, Irvine, CA, USA) [34]. Subsequently, the introduction of the SAPIEN 3 Ultra RESILIA (S3UR) prosthesis, thanks to its inclusion of a 40% higher sealing skirt in addition to an outer textured polyethylene terephthalate skirt, has further led to improvement in paravalvular leak degree [35].

Unfortunately, similar results have not been achieved in ViV procedures yet. Dimitriadis et al., in their recent systematic review and meta-analysis based on an extremely large population (17,581 patients included, 9163 undergoing ViV-TAVI and 8418 undergoing redo-SAVR), highlighted the presence of a significantly higher risk of paravalvular regurgitation in the ViV-TAVI group compared to the redo-SAVR group (RR: 2.44; 95% CI: 1.73 to 3.45) [24]. Similarly, Gatta et al., in a multicenter UK retrospective study, demonstrated a high degree of aortic regurgitation already at hospital discharge in patients undergoing ViV-TAVI (redo SAVR 1.6% vs. 19% in patients undergoing ViV-TAVI, *p* value < 0.001) [25].

In light of these extremely consistent data, there is a need to identify the real risk factors for paravalvular leak, especially in patients with a high life expectancy.

**Figure 3:**
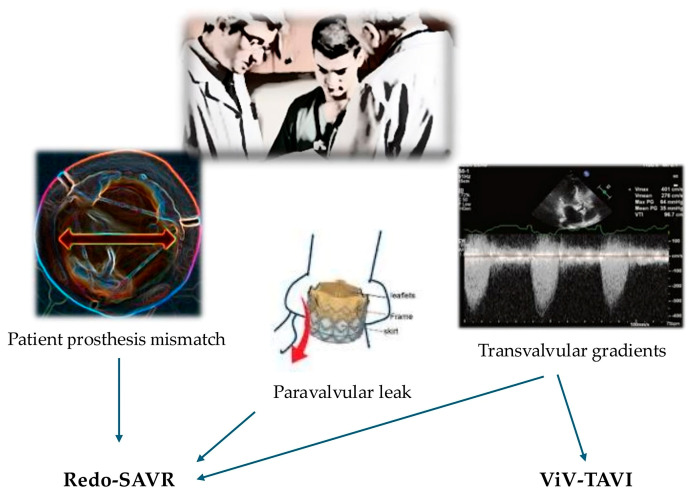
Summary of choice between redo-SAVR and ViV-TAVI based on hemodynamic aspects.

Figure 3 summarizes how hemodynamic aspects influence the decision to employ redo-SAVR vs. ViV-TAVI.

### 3.2. ViV-TAVI vs. Redo SAVR: Technical Aspects

#### 3.2.1. Coronary Obstruction

Coronary obstruction is one of the most feared complications of ViV-TAVI, associated with a high mortality rate [36]. From the ViVID registry emerges an incidence rate of 2.3%, approximately four times higher than that for the control group, and a high 30-day mortality rate (48.6% vs. 3.7% with *p* < 0.001) [37].

Based on these data, and considering that in almost all the cases, the coronary obstruction is caused by displacement of the bioprosthetic leaflets towards the coronary ostium (more frequently in stentless valves and stented bioprostheses with externally sutured leaflets) [37,38], it is always necessary to identify anatomical risk factors in addition to the standardization of the implantation techniques.

Among the most important anatomical aspects for predicting the risk of coronary obstruction is the distance between the prosthesis implant site and the coronary ostia, the virtual-THV (transcatheter heart valve)-to-coronary-ostial distance (VTC).

The supra-annular implant and the different orientation of the previously positioned valve prosthesis can further reduce the VTC; therefore, post-processing evaluation by cardiac CT (Computed Tomography) of the correct distance is fundamental in evaluating the risk of coronary obstruction. The Vancouver approach is one of the most widely used methods in the preoperative study phase, simulating the presence of a cylinder with a defined diameter and height representing the expanded prosthesis, with the center of the cylinder aligned with the center of the basal ring. Furthermore, a cut-off distance of 4 mm was obtained through a centralized CT laboratory evaluation of 20 patients with coronary obstruction and 90 consecutive controls from two centers [37].

Similarly, it is useful to use coplanar projections characterized by an RAO (Right Anterior Oblique) and LAO (Left Anterior Oblique) angle and a corresponding cranial or caudal angle to guide coaxial positioning [39].

Furthermore, the use of BASILICA-type devices (bioprosthetic or native Aortic Scallop Intentional Laceration to prevent iatrogenic coronary artery obstruction during TAVR) has shown increased procedure safety with both native and prosthetic valves. In fact, the BASILICA trial showed a 100% success rate, with no cases of coronary obstruction and reintervention, and rare cases of hemodynamic instability resolved after TAVI prosthetic implantation [40].

Another more recent device that guarantees adequate protection of the coronary ostia is the ShortCut device (Pi-Cardia). It is designed to separate the leaflets of the previously implanted bioprosthesis from the coronary ostia in order to increase the size of the opening to the coronary flow by splitting the aortic leaflets through a controlled device [41]. The Short-Cut study demonstrated successful leaflet splitting in the entire population (sixty patients) and a rate of 30-day freedom from coronary obstruction of 95% (95% CI 86.1–99.0%) [42].

Therefore, it is clear that preoperative CT scan evaluation is essential in order to reduce the risk of coronary obstruction and, if the risk of coronary obstruction is present, the use of coronary protection procedures and devices.

The problem of coronary obstruction does not concern redo-SAVR procedures, as in most cases, this technique does not involve the reconstruction of the aortic root, and therefore the possible replanting of the coronary ostia.

#### 3.2.2. Implantation Techniques

In 2007, the technical feasibility of the ViV procedure was studied in pigs with the use of transapical access to deploy 23 mm Cribier–Edwards transcatheter valves into Carpentier–Edwards valves in the aortic and mitral positions [6,43]. In 2010, the hemodynamic function of TAVI was studied in vitro in a series of experiments implanting 23 mm SAPIEN valves in variously sized Carpentier–Edwards PERIMOUNT valves [44,45] and, later, 23 and 26 mm Evolut R valves in variously size Hancock II valves [6,46].

The first clinical transcatheter ViV case was reported by Wenaweser et al. in 2007 and used a self-expanding Medtronic CoreValve [6,47]. In 2015, transcatheter ViV was approved by the U.S. Food and Drug Administration (FDA) for use in the aortic position with both the CoreValve and SAPIEN XT valves [6,48].

Among the main technical difficulties of ViV-TAVI is the challenge of correctly anchoring the prosthesis into the previously implanted valve, especially in the case of stented prostheses, in which, in addition to a radiopaque marker that identifies the correct positioning of the prosthesis, there is also a frame or stent to which it can be anchored. Furthermore, it has been observed that correct prosthesis positioning is more difficult in the case of a previous bioroot (as in the case we treated) rather than a prosthesis with calcific degeneration [49]. For these reasons, in the preoperative phase, multimodal reconstruction using cardiac CT plays a crucial role in this case.

In addition to differences in patient anatomy, the TAV used influences postoperative hemodynamic performance. ViV-TAVI is performed using self-expanding or balloon-expandable valves [6]. The choice of a self-expandable versus a balloon-expandable valve depends on several factors: patient anatomy, distance from the coronary ostia and the characteristics of the previously implanted prosthesis [49].

The 1-year results of the LYTEN Trial, based on a population of 98 patients with dysfunction of a biological prosthesis with a size ≤ 23 mm who underwent ViV-TAVR, among which approximately 47% underwent implantation of a balloon-expandable prosthesis and 53% underwent implantation of a self-expandable one, showed more hemodynamically satisfactory results, in terms of lower transvalvular gradients and a higher indexed orifice effective area, in patients with a self-expanding prosthesis, thanks to its supra-annular positioning [50]. The improved hemodynamic performance could also explain the higher rate of moderate or severe deterioration in the case of balloon-expandable devices [51].

Conversely, in the presence of very rigid aortic prostheses that require high pressures for their fracture, such as Perimount and Magna Ease (Edwards Lifesciences), better results are obtained, in terms of better anchoring and absence of deformation, when using balloon-expandable prostheses, which are characterized by greater radial force compared to self-expanding ones [52]. However, at the same time, this characteristic can turn into a contraindication to the use of balloon-expandable prostheses in cases of particularly stubborn aortic calcifications that increase the risk of annular rupture [53].

Finally, another extremely important aspect is the evaluation of VTC, as previously discussed. When this parameter is below the safety range, the use of expandable balloon prostheses is certainly advisable.

Percutaneous femoral artery access is the most common approach for TAVR and ViV procedures in the aortic position [6,54]. Alternative accesses for aortic ViV procedures are transapical, direct aortic, subclavian and carotid artery approaches [6].

In the case of ViV TAVI within a conduit, such as the Biointegral conduit, different anatomical aspects must be considered. The Biontegral conduit is a stentless porcine aortic root sewn to a woven vascular graft for the ascending aorta; therefore, the annulus does not have a rigid structure (plastic or metal) to facilitate anchoring of the TAV, and it does not have a radiopaque marker. Furthermore, the possibility of displacement of the aortic leaflets towards the coronary ostia is higher since they are reimplanted. In addition, this risk is much higher as the prosthesis is stentless and therefore has more mobile leaflets [55].

**Figure 4:**
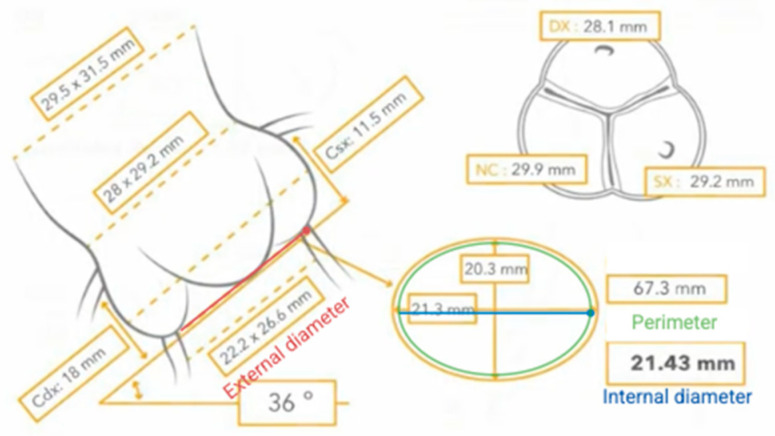
Differences between external and internal diameter.

Another important anatomical aspect, especially for correct sizing, is the difference between the external and internal diameter, due to the internal insertion of the leaflets. In fact, it has been shown that external insertion of the leaflets results in a reduction in the internal diameter of approximately 2 mm [56], as in the case we are reporting (Figure 4).

It is clear that performance of the ViV TAVI procedure on a degenerated Biointegral conduit is linked to greater complexity, and therefore requires greater operator experience.

Similarly to as highlighted in the case of ViV-TAVI, in redo-SAVR procedures, preoperative evaluation using multimodal cardiac CT techniques plays a fundamental role in understanding the relationships of the aortic and cardiac structures with the chest wall in order to limit, as much as possible, complications related to re-sternotomy. Furthermore, correct planning must also be conducted for the cannulation strategy in order to ensure the best peripheral perfusion, reduce the risk of embolization and allow the establishment of circulatory arrest if intraoperative complications make it necessary [57]. Finally, myocardial and cerebral protection techniques and pharmacological treatments to minimize the risk of bleeding in the perioperative period constitute the final piece of a surgical strategy that, in cases of redo-SAVR, requires careful planning and the cooperation of multiple professionals, such as cardiac anesthetists, perfusion technicians and theater nurses.

Again, the choice of prosthesis depends on the evaluation of numerous aspects: the patient’s age and life expectancy, the type of prosthesis previously implanted and the surgery performed (single-valve replacement or aortic root reconstruction), and the anatomy of the aortic root.

Age and life expectancy influence not only the choice between a biological or mechanical prosthesis, but also the possibility of a subsequent ViV-TAVI, for which prostheses that can guarantee a larger effective orifice area should be preferred.

In the presence of small biological prostheses, surgical root enlargement strategies such as the Nicks, Manouguian, Konno–Rastan or Yang techniques may be necessary in the reintervention procedure. Indeed, the use of small biological prostheses has a significant effect on determining PPM, but more importantly, it has been shown that aortic root enlargement procedures do not statistically significantly increase mortality and the rate of major cardiovascular events compared to redo SAVR without root enlargement [58]. Sometimes, in the case of extensive calcifications or previous pericardial patch grafting, it may be necessary to replace the entire aortic root with coronary ostia reimplantation [59]. Finally, in patients at high surgical risk who therefore require a short cardiopulmonary bypass and aortic cross clamp time, the use of sutureless prostheses certainly represents an advantage for the subsequent postoperative outcomes [60].

**Figure 5:**
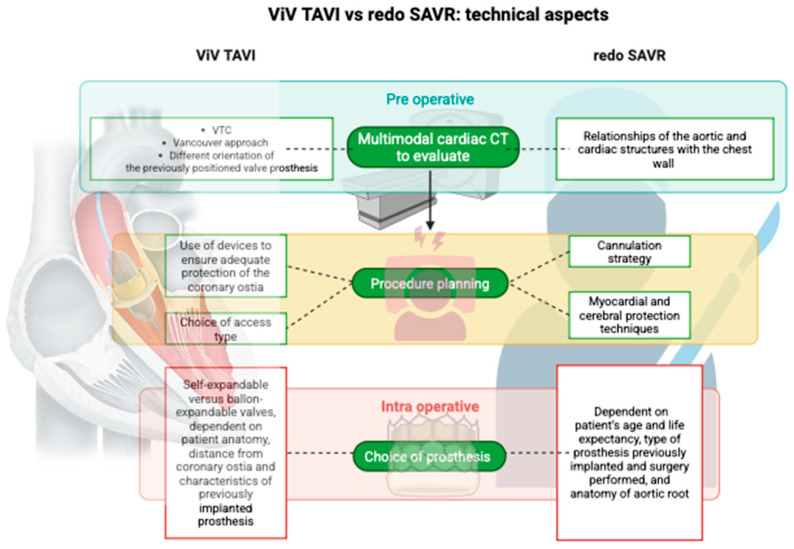
Different technical aspects between ViV TAVI and redo SAVR.

From what has been discussed, it is clear that many variables need to be considered when dealing with redo SAVR or ViV-TAVI (Figure 5).

## 4. Conclusions

Increasing life expectancy and the growing use of biological prostheses make it necessary to improve reintervention techniques. The prohibitive surgical risks of some patients have led to the progressive development of ViV-TAVI techniques as an alternative to redo-SAVR. However, the hemodynamic aspects of this approach are the source of strong controversy in the choice of the most adequate procedure: the evaluation of transvalvular gradients can, from time to time, make us lean towards one or the other procedure, unlike the presence of paravalvular leak and patient–prosthesis mismatch, which, in most cases, have a lower hemodynamic impact in the case of redo-SAVR compared to in the case of ViV-TAVI. A review of technical aspects shows that personalization of the type of treatment based on preoperative imaging evaluation is essential for the success of the procedure. Finally, while in the literature there are significant numerical data for the use of redo-SAVR and ViV-TAVI with degenerated biological prostheses, the same cannot be said for the degeneration of ducts, such as biointegral ducts, since these procedures are associated with a greater degree of complexity. Significant limitations to our study include the restricted number of centers that deal with ViV-TAVI compared to those that deal with TAVI. This restriction, due to the greater difficulties in the execution of the ViV-TAVI procedure, leads to lower availability of data and a lower impact of the work of meta-analyses and reviews such as ours. In particular, most of the studies analyzed are observational studies that entail bias due to the design of the study. Therefore, it is desirable, first of all, for us to achieve standardization of the technique through greater use of defined imaging techniques and increasingly appropriate choices of the devices used. This could lead to a greater number of multicenter registries with consequent improvements in the analysis of performance and survival rates, especially in consideration of the growing complexity of valve technologies and differences in experience among centers.

## Figures and Tables

**Table 1:** Studies with the most significant aspects for our review.

Authors	Year	Study Population	Advantages of ViV Over Redo SAVR	Disadvantages of ViV Over Redo SAVR
Nalluri N. et al. [18] [systematic review and meta-analysis]	2018	594 patients: 255 underwent ViV-TAVI and 339 underwent redo-SAVR	No statistically significant differences in post-procedural transvalvular gradients Lower risk of permanent pacemaker implantation, life-threatening complications and major bleeding	Significantly higher risk of paravalvular leak and vascular complications
Gozdek M. et al. [19] [systematic review and meta-analysis]	2018	342 patients: 176 underwent ViV-TAVI and 166 underwent redo-SAVR	Lower risk of permanent pacemaker implantation and bleeding events	High risk of vascular complications and readmissions Less satisfactory hemodynamic results: higher mean postoperative aortic valve gradient and greater risk of postoperative aortic valve gradient (>20 mmHg)
Dokollari A. et al. [20] [single center study]	2021	88 patients: 31 underwent ViV-TAVI and 57 underwent redo-SAVR	No differences in post-procedural transvalvular gradients Shorter hospital stays, less need for red blood cell transfusions Lower risk of permanent pacemaker implantation at 3-year follow-up	Higher incidence of postoperative regurgitation
Spaziano M. et al. [21] [multicenter study]	2017	205 patients: 79 underwent ViV-TAVI and 126 underwent redo-SAVR	Shorter length of hospital stay	Higher mean postoperative aortic valve gradient and incidence of postoperative > 20 mmHg aortic valve gradient
Raschpichler M. et al. [22] [systematic review and meta-analysis]	2022	8881 patients: 4458 underwent ViV-TAVI and 4423 underwent redo-SAVR	Better short-term mortality	Greater risk of prosthetic aortic valve regurgitation and severe patient–prosthesis mismatch Higher mean postoperative aortic valve gradient
Tam D.Y. et al. [23] [systematic review and meta-analysis]	2020	558 patients: 214 underwent ViV-TAVI and 344 underwent redo-SAVR	Lower 30-day mortality rates, less permanent pacemaker implantation and shorter postoperative hospital stay	//
Dimitriadis K. et al. [24] [systematic review and meta-analysis]	2025	17581 patients: 9163 underwent ViV-TAVI and 8418 undewent redo-SAVR	Lower 30-day and 1-year mortality rates Less frequent major bleeding events and permanent pacemaker implantations	Higher mean transprosthetic gradient, rates of severe patient–prosthesis mismatch and paravalvular leak
Gatta F. et al. [25] [multicenter retrospective study]	2023	1322 patients: 911 patients underwent redo-AVR and 411 patients underwent Valve-in-Valve TAVI	Lower mortality rates Lower frequency of major postoperative complications	Higher mean transprosthetic gradients and grade of paravalvular leak

## Supplementary Materials

The following supporting information can be downloaded at https://www.mdpi.com/article/10.3390/jcm14207158/s1. Video S1: CoreValve Evolut FX 23 mm in degenerated 25 mm Biointegral conduit procedure.
